# Deregulation of Frizzled Receptors in Hepatocellular Carcinoma

**DOI:** 10.3390/ijms19010313

**Published:** 2018-01-21

**Authors:** Kristy Kwan-Shuen Chan, Regina Cheuk-Lam Lo

**Affiliations:** 1 Department of Pathology, Li Ka Shing Faculty of Medicine, The University of Hong Kong, Hong Kong, China; kristyks@pathology.hku.hk; 2 State Key Laboratory for Liver Research, The University of Hong Kong, Hong Kong, China

**Keywords:** hepatocellular carcinoma, G protein-coupled receptors, Frizzled receptors, Wnt signaling

## Abstract

G protein-coupled receptors (GPCRs) have a substantial role in tumorigenesis and are described as a “cancer driver”. Aberrant expression or activation of GPCRs leads to the deregulation of downstream signaling pathways, thereby promoting cancer progression. In hepatocellular carcinoma (HCC), the Wnt signaling pathway is frequently activated and it is associated with an aggressive HCC phenotype. Frizzled (FZD) receptors, a family member of GPCRs, are known to mediate Wnt signaling. Accumulating findings have revealed the deregulation of FZD receptors in HCC and their functional roles have been implicated in HCC progression. Given the important role of FZD receptors in HCC, we summarize here the expression pattern of FZD receptors in HCC and their corresponding functional roles during HCC progression. We also further review and highlight the potential targeting of FZD receptors as an alternative therapeutic strategy in HCC.

## 1. Introduction

The G protein-coupled receptors (GPCRs) belong to a superfamily that can be activated by a diverse range of ligands [1,2]. Being a cell surface receptor, their activation mediate downstream molecular signaling cascades leading to the regulation of cellular functions. Studies on GPCR-associated tumorigenesis have further revealed the role of GPCRs as a “cancer driver” during tumor development [2,3,4,5,6]. The GPCRs comprise the largest class of membranous proteins in the human genome. Its family members are categorized into five clusters according to their sequence similarities within the seven-transmembrane (7-TM) region [7]. These include the rhodopsin family, the adhesion family, the Frizzled (FZD) family, the glutamate family, and the secretin family. The 7-TM GPCRs have a signature of coupling with G proteins to activate downstream signaling pathways. G proteins consist of two classes, namely heterotrimeric G proteins and monomeric G proteins. The heterotrimeric G proteins are an intracellular complex containing three subunits: Gα, Gβ, and Gγ. Agonist binding induces a conformational change of the GPCR that subsequently recruits GDP-bound G proteins and the G protein is activated by the exchange of guanosine disphosphate (GDP) to guanosine triphosphate (GTP). The GTP-bound α subunit is dissociated from the βγ complex, which in turn activates its respective effectors. The monomeric G proteins, also known as small G proteins, are homologous to the α subunit. Similar to the α subunit, upon ligand stimulation, activation of the small G proteins follows the GTPase cycle to transduce the signal to downstream effects. The GPCR-related signaling pathways include the Rho/Rho-associated kinase (ROCK) pathway, the c-Jun N-terminal protein kinase (JNK)/stress-activated protein kinase (SAPK) pathway, the Hippo pathway, and Wnt signaling [2]. Notably, the above signaling pathways have been heavily implicated in tumorigenesis, indicating the significance of GPCRs in cancer development. Among the common human cancers, hepatocellular carcinoma (HCC) is tightly associated with canonical Wnt signaling in which FZD receptors are intrinsic key players. Potential downstream pathways of Wnt/FZD signaling is reported to be activated in nearly 90% of analyzed HCC cases, suggesting that Wnt/FZD signaling is an important pathway in HCC development [8]. Deregulation of Wnt pathway is mainly due to the *CTNNB1* mutation and upregulation of Wnt/FZD expression. Since FZD receptors belong to GPCRs and Wnt signaling has an indispensable role in HCC development, we aim in this article to summarize the up-to-date information on deregulation of FZD receptors in HCC and review the therapeutic implications of FZD receptors in HCC.

## 2. Basics of Frizzled Proteins

FZDs are 7-TM receptors that are categorized as class FZD of the GPCRs [9,10]. To date, ten FZD members—FZD 1 to 10—have been identified within the FZD family in humans. Basically, they are divided into four clusters based on their amino acid identity. These include cluster 1 (FZD1/2/7), cluster 2 (FZD5/8), cluster 3 (FZD4/9/10), and cluster 4 (FZD3/6) [11]. Structurally, FZD receptors comprise an extracellular N-terminus, three extracellular and three intracellular loops together with an intracellular C-terminus [12,13]. FZDs are known to be receptors for various ligands including the Wnt proteins, R-spondin, and Frizzled-related proteins. To activate signal transduction, the extracellular N-terminus contains a conserved cysteine-rich domain (CRD) that serves as a primary site for ligand binding, while the intracellular C-terminus consists of a conserved juxtamembrane motif (KTxxxW) for binding with proteins containing a PDZ (Psd-95/Disc large/ZO-1 homologous)-binding domain. FZDs are widely expressed across various tissues and the pattern is different between adult tissues and embryonic tissues [14,15,16], suggesting that they have distinct functional roles during biological processes. FZD1 to 8 are expressed in adult mouse liver with FZD2/4/6/7/8 being expressed in hepatocytes; FZD1/2/3/4/6/7/8 are expressed in biliary epithelial cells; FZD2/3/4 and FZD6/7/8/9 are expressed in stellate and Kupffer cells, and FZD1/2/3/4/6/7/8 are expressed in hepatic sinusoidal endothelial cells [16]. Since Wnt signaling is one of the utmost important signaling pathways in both developmental processes and carcinogenesis, the Wnt/FZD signaling cascade will be further discussed.

## 3. Role of the Wnt/FZD Signaling Cascade in Liver Physiology

Wnt signaling is known to be essential for embryogenesis and maintenance of tissue homeostasis [17]. To activate intracellular signaling transduction, extracellular Wnts bind to FZD receptors and low-density lipoprotein (LDL) receptor-related proteins 5 and 6 (LRP 5/6) [18]. Three types of Wnt signaling cascades are defined by the downstream effector players upon stimulation of Wnts: canonical Wnt/β-catenin signaling, non-canonical Wnt signaling, and Wnt/Ca^2+^ signaling. The major difference among them is the involvement of β-catenin. Among all, the canonical Wnt signaling is the most characterized. In the absence of Wnts, cytoplasmic β-catenin undergoes proteasomal degradation that is governed by a “destruction complex” containing Axin, adenomatous polyposis coli (APC), glycogen synthase kinase 3β (GSK3β), and casein kinase 1 (CK1) [18,19]. As a result, β-catenin is maintained at a low level, leading to the low activity of this pathway. In the presence of Wnts, they interact with FZD receptors and LRP 5/6, leading to the recruitment of Dishevelled (DVL) proteins and G proteins. These proteins further recruit Axin to the plasma membrane and the “destruction complex” is disrupted. Therefore, cytoplasmic β-catenin is stabilized and accumulates in the cells. Accumulated unphosphorylated β-catenin translocates to the nucleus and eventually binds to the lymphoid enhancer factor/T cell factor (LEF/TCF) transcription factors to exert effects on downstream gene transcription. For the non-canonical Wnt signaling, also known as the planar cell polarity (PCP) pathway, its signal transduction is also activated with the involvement of Wnt/FZD interaction and activation of the small G proteins [20,21]. This cascade relies on the core proteins including DVL, VANGL 1/2, CELSR1, and Prickle 1/2 to activate the downstream Rho/ROCK and JNK pathway to regulate cell polarity. In the Wnt/Ca^2+^ pathway, intracellular storage of Ca^2+^ is released upon the activation of phospholipase triggered by the Wnt/FZD interaction, and G proteins have a critical role in inducing the downstream cascade through interplay between the FZD receptors [21]. Upon stimulation of Wnts, FZD receptors activate heterotrimeric G proteins, which is followed by the activation of phospholipase C. Subsequently, the Ca^2+^/NFAT pathway is activated, leading to target gene transcription. The downstream effectors upon stimulation of Wnts vary; however, they all share the same upstream complex—Wnt/FZD interaction.

Wnt/β-catenin signaling is heavily implicated in liver biology. It has a dynamic activity throughout liver development, which is determined by the upstream effectors—Wnt/FZD receptor interaction. This pathway has a functional role throughout prenatal liver development and is regulated for early liver formation, liver size, and liver lineage differentiation [22,23]. During prenatal liver development, 13 out of 19 Wnts and all 10 FZD receptor genes have been detected [24]. Repression of Wnt/β-catenin signaling is required for liver formation from organ buds [25,26], while activation of Wnt/β-catenin signaling is responsible for liver size of the developing liver by regulating cell proliferation and apoptosis [27,28,29]. Furthermore, studies have revealed that its activation is essential in controlling cell lineage during liver development [27,29,30]. This indicates that Wnt/β-catenin signaling is one of the important pathways during prenatal liver development, although a more detailed molecular mechanism needs to be defined. In postnatal liver development, Wnt/β-catenin signaling appears to bear a more critical function in liver regeneration and metabolism. Upon liver injury induced by partial hepatectomy and acetaminophen in mice, nuclear β-catenin was found to be an element required for liver regeneration [31,32,33]. Moreover, hepatic progenitor cells, a population of quiescent cells that reside in the healthy liver, have the ability to differentiate into hepatocytes through Wnt signaling in response to liver injury [34]. This indicates that the Wnt/β-catenin pathway promotes liver regeneration. The liver is an organ with characteristic metabolic zonation. The zones are generally referred to as the periportal (area of the portal triad) and perivenous zones (area of the central vein). Multiple independent studies have revealed that the Wnt/β-catenin pathway contributes to the regulation of perivenous hepatocyte gene expression (glutamine synthetase) and the nuclear transcription factor hepatocyte nuclear factor-4α [35,36,37,38]. There is a phenotypic difference between perivenous and periportal hepatocytes as they exert opposite metabolic functions according to the oxygen gradient [23,39]. The higher activity of Wnt/β-catenin signaling restricted in the perivenous hepatocytes suggests that Wnt/β-catenin signaling is important for the metabolic function dominated in this zone. The above summarized the role of Wnt/β-catenin signaling in liver development and metabolic functions.

## 4. Canonical Wnt/β-Catenin Signaling in Hepatocellular Carcinoma (HCC)

Liver cancer is one of the deadliest cancers worldwide of which HCC is the major form [40,41]. Development of HCC involves multiple steps from liver inflammation to hepatic fibrosis to cirrhosis to HCC. Accumulation of multiple gene mutations and aberrant signaling pathways are known to be frequently altered during HCC pathogenesis. Of all, Wnt/β-catenin signaling is suggested to be a major driver pathway in HCC, while the non-canonical Wnt signaling is less detailed in HCC [8,42,43]. A portion of HCC cases demonstrate hyperactivated Wnt/β-catenin signaling. The hyperactivation has been linked to genetic mutations related to this pathway, including *CTNNB1* (11–37% of HCC cases), *AXIN1* (10% of HCC cases), *APC* (less than 2% of HCC cases), and *ZNRF3* (3% of HCC cases) [41]. Approximately 40–70% of HCCs harbor accumulation of β-catenin in the nucleus, which is a hallmark of the activation of the canonical Wnt/β-catenin signaling [44]. This suggests that activation of the Wnt/β-catenin pathway in a portion of HCCs is attributed to non-mutational dysregulation of the upstream components along the pathway. Apart from mutational events, the altered expression of key players of these pathways—for example, Wnts, FZD receptors, and the secreted Frizzled-related protein (sFRP)—was found to regulate the pathway activity [8]. In particular, a study has revealed that upregulation of FZD7 is correlated with increased expression of β-catenin target genes in HCC with wild-type β-catenin, suggesting that activation of Wnt/β-catenin signaling can be a consequence of deregulation of the FZD receptors [45]. Multiple independent studies have revealed the mechanism underpinning the activation of Wnt/β-catenin signaling with regard to the well-known risk factors of HCC. Although *CTNNB1* mutation is more significant in hepatitis C virus (HCV)-related HCC and aflatoxin B1-related HCC as compared to hepatitis B virus (HBV)-related HCC [41,43,46], a recent study revealed that genetic polymorphisms in *CTNNB1* and *AXIN1* are correlated to HBV-related HCC development and patients’ survival outcomes [47]. Furthermore, accumulating evidence has shown that hyperactivation of Wnt/β-catenin signaling in HBV-related HCC is related to hepatitis B X protein (HBx) [48,49,50,51].

Activation of Wnt/β-catenin signaling is responsible for HCC development and cellular differentiation [52]. An early study documented the positive correlation between dysregulation of Wnt/FZD events and poor tumor differentiation status in clinical HCC tissues [8]. A recent study has dissected the gene network in HCC containing fibrous nests. Analyses revealed that HCC cells with high expression of α-smooth muscle actin harbor a high level of cell surface Wnt pathway components, including FZD1 and 7, and tumor dedifferentiation [53]. Pieces of evidence have demonstrated that Wnt/β-catenin signaling contributes to HCC development by facilitating cancer cell proliferation, cancer cell migration, epithelial-to-mesenchymal transition (EMT), and inducing stemness of cancer cells [54,55,56,57]. These indicate that Wnt/β-catenin signaling confers the aggressive phenotype of HCC cells. Since Wnt/β-catenin signaling involves three complexes—the Wnts/FZD receptor complex, the cytoplasmic “destruction complex”, and the nuclear β-catenin/TCF/LEF complex—the activation of this pathway can be induced at multiple points along this cascade. To review the significance of FZD receptors in HCC, we focus on and discuss the FZD receptors below regarding their expression, mechanisms, and therapeutic implications in HCC.

## 5. Deregulated Expression and Roles of FZDs in HCC

FZD receptors are known to be receptors for Wnts and they are the mediators that induce the downstream signaling pathway. Since canonical Wnt signaling is frequently activated in HCC, it is not surprising that FZD receptors have an indispensable role in this pathway. In an FZD receptor-centric view of Wnt signaling, Wnts interplay with FZD receptors and subsequently deactivate the “destruction complex”. Ultimately, stabilized β-catenin accumulates in the nucleus to induce transcriptional regulation. In this regard, the deregulated expression of FZD receptors has a substantial effect on the canonical Wnt pathway in HCC through modulation of the “destruction complex”. A comprehensive study reported the expression pattern of FZD receptors in clinical HCC samples [8]. FZD3/6/7 was analyzed to be frequently upregulated at both the transcript and protein levels in HCC tissues while expression of the remaining members FZD1/2/4/5/8/9/10 is not significantly altered. Specifically, FZD3/6/7 was reported to be upregulated in 30% to 40% of analyzed HCC tissues when compared to normal liver tissues. Strikingly, a higher expression of FZD3/6/7 has been detected in HCC cells compared to normal hepatocytes by immunostaining. These further shortlist the potential players of FZD receptors in HCC progression. Recently, two independent studies reported that FZD2 mRNA level is overexpressed in HCC and its upregulation is correlated to advanced stages of HCC and poorer recurrence-free survival, respectively [58,59]. In these studies, FZD2 expression is correlated to a more mesenchymal phenotype of HCC. Functionally, FZD2 induces HCC cell migratory ability and invasiveness, as well as EMT, indicating that FZD2 appears to be an oncogene in HCC. In another study, the expression of FZD1/7 was reported to be significantly higher in HCC with a high grade of fibrous hotspots [53]. HCC containing fibrous nests is less differentiated, further suggesting that FZD receptors are correlated to a more aggressive HCC phenotype. Still, further verification with clinical samples is warranted. Among all FZD receptors, FZD7 is relatively more characterized in HCC. Upregulation of FZD7 has been detected in both HCC and surrounding dysplastic liver tissues and its upregulation has been correlated to increased protein expression of β-catenin in both murine and human HCC cells [45,60,61]. The expression status of FZD receptors in HCC tissues is summarized in Table 1.

To date, although there is scarce information on the correlation between FZD7 and clinicopathological features in HCC, reports have shown that FZD7 exerts its functional role on HCC progression through enhancing HCC cell motility, cell growth, and stemness properties in vitro [62,63,64]. Having said that, while the expression pattern of FZD3/6 has been revealed in clinical HCC, their functional roles in hepatocarcinogenesis remain largely unknown. To date, one report has postulated that upregulation of FZD6 contributes to HCC progression in a genetic mouse model [65]. Further detailed functional roles of FZD3/6 await to be explored. Apart from the abovementioned FZD receptors, the functional roles of FZD4/9 were characterized in a human HCC cell line model. Data suggest that FZD4/9 enhances HCC cell proliferation and cell motility in vitro while FZD4 might also confer cancer stemness properties in HCC [66,67]. All in all, FZD1/2/3/6/7 has been reported to be upregulated in HCC while FZD2/4/6/7/9 confers a more aggressive HCC phenotype.

## 6. Mechanism of Deregulated FZDs in HCC

**Figure 1:**
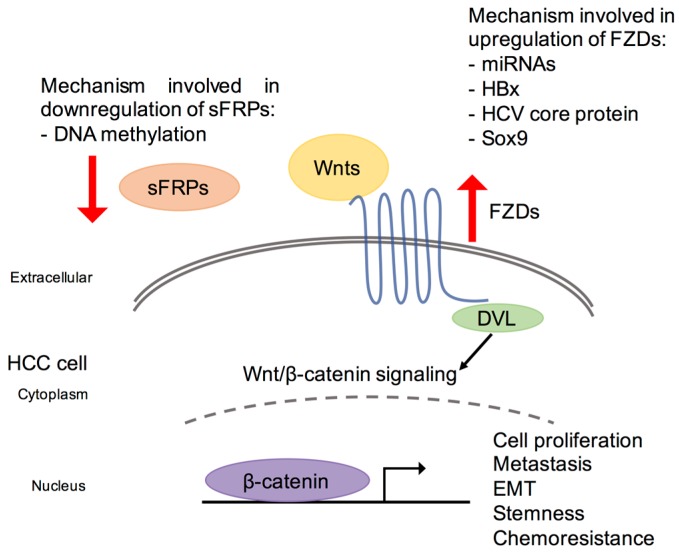
Schematic diagram of deregulated frizzled (FZD) receptors in the Wnt/β-catenin signaling pathway in hepatocellular carcinoma (HCC). Wnt/β-catenin signaling pathway is frequently activated in HCC. In terms of deregulated activation of FZD receptors, the underlying mechanism includes downregulation of sFRP1/2/5 as a result of DNA hypermethylation detected in HCC and upregulation of FZD receptors as a consequence of miRNA regulation, hepatitis B X protein (HBx) and hepatitis C virus (HCV) core protein involvement and overexpression of transcription factor Sox9. DVL, dishevelled; EMT, epithelial-to-mesenchymal transition; FZD, frizzled; HBx, hepatitis B X protein; sFRPs, secreted frizzled-related proteins.

In view of the frequent activation of Wnt signaling pathway in HCC progression, FZDs have an apparent role in modulating this pathway as they serve as upstream regulators of the Wnt signaling pathway. Generally, upregulation of FZDs lead to the activation of Wnt/β-catenin pathway and, to a lesser extent, the non-canonical pathway [45,58,60,61,64]. The mechanism involved in the deregulation of FZDs in HCC will be reviewed in terms of upstream regulators and downstream molecular events. A schematic diagram is presented in Figure 1, highlighting the mechanism involved in the deregulated activation of FZD receptors in HCC.

sFRPs are antagonists of Wnt pathway through direct interaction with Wnts [17]. In view of their inhibitory role in Wnt signaling, they serve as an upstream regulator and are related to the activation status of Wnt/FZD signaling. sFRP1/2/5 was reported to be frequently hypermethylated in HCC, suggesting that downregulation of these sFRPs contribute to HCC carcinogenesis through activation of Wnt signaling [68]. Among all, sFRP1 has been relatively well characterized in HCC. sFRP1 is identified as a tumor suppressor gene and its expression can be restored by 5-aza-2′deoxycytidine DNA demethylation treatment in HCC [69,70,71,72]. As reported by three independent studies, suppression of sFRP1 induces EMT through Wnt/β-catenin signaling in vitro and leads to HCC growth and lung metastasis in vivo, respectively [71,73,74]. Coherently, restoration of sFRP1 expression could be achieved by DNA demethylation and exogenous sFRP1 reverses the functional effects observed indicating that downregulation of sFRP1 through DNA hypermethylation augments Wnt/β-catenin signaling in HCC. Besides, sFRP1/3/5 was revealed to be downregulated in two independent HCC cohorts [75,76]. Given the mechanism between sFRPs and Wnt/FZDs on the alteration of the Wnt signaling pathway, downregulation of sFRPs in HCC suggests that Wnt signaling is activated in response to Wnt/FZD binding.

Another upstream regulator that governs Wnt/FZD signaling is microRNAs (miRNAs). Accumulating evidence has shown that FZDs are partially regulated by miRNAs. FZD7 was found to be a target of miR-27a and miR-199a; the negative correlation between FZD7 and miR-199a was validated in clinical HCC tissues [63,77]. FZD6 is a target of miR-194 and the inverse correlation was confirmed in a genetic mouse model of HCC [65]. Furthermore, underexpression of Let7b induces cancer stemness in HCC, cell proliferation, migration, and invasion through activation of Wnt signaling via the upregulation of FZD4 in an HCC cell line model [67]. Regulation of miRNAs on other FZDs await investigation.

HBV and HCV are the major risk factors for HCC. They contribute to HCC development largely through the induction of genetic instability in HBV DNA integration, HBx, and HCV core proteins [78]. By cDNA microarray and in vitro experiments, it has been revealed that HBx is associated with the upregulation of FZD10 in a transformed liver cell line with HBx [79,80]. For HCV-related HCC, the HCV core protein induced Wnt pathway components, including the expression of FZD1/2/5/6/7/9, and further activated β-catenin/TCF transcription activity in an HCC cell line [81]. This implies that HBV and HCV promote HCC development through modulation of Wnt/FZD signaling. Validation with clinical HCC samples will further consolidate the linkage between them.

Wnt/FZD stays upstream of Wnt signaling, indicating that alterations in FZD affect the downstream signaling cascade. Indeed, we previously reported that Sox9 is a novel regulator of FZD7 in HCC and delineated that overexpression of Sox9 confers stemness and chemoresistance in HCC through FZD7-mediated Wnt/β-catenin signaling [64]. Furthermore, the positive correlation between Sox9 and FZD7 was validated in clinical HCC samples. Multiple Wnt target genes related to stemness are downregulated upon silencing of Sox9 in HCC cell lines, suggesting that FZD7-mediated Wnt/β-catenin signaling is responsible for the induction of stemness genes in HCC. Another independent study demonstrated that β-catenin, c-Myc, and cyclin D1 are suppressed upon knocking down FZD7 in HCC cell lines [82]. This implies that FZD7-mediated Wnt/β-catenin signaling regulates HCC cell proliferation through c-Myc and cyclin D1. FZD2 was reported to be upregulated in HCC tissues. Functionally, overexpression of FZD2 has conferred that EMT in HCC is dependent on non-canonical Wnt signaling instead of Wnt/β-catenin signaling [58]. The study elegantly demonstrated that Wnt5/FZD2 induces EMT in HCC through phosphorylation of Fyn and Stat3. This data uncovered the role of FZD2 in HCC through a less well-characterized non-canonical pathway.

## 7. Therapeutic Implications of FZDs in HCC

Given that activation of Wnt signaling confers HCC aggressiveness, the Wnt signaling components appear to be attractive targets for therapeutic interventions in HCC. Among all 10 FZD receptors, FZD7 is suggested to be an emerging target due to the fact that its expression is upregulated in over 60% of HCC tissues [8,45,60,61]. To assess the efficacy of suppressing HCC growth through targeting FZD7, small interfering peptides have been constructed to block the interaction between FZD7 and DVL [83]. Results demonstrate that targeting FZD7 induces HCC cell apoptosis through the degradation of β-catenin. Mechanistically, the functional small interfering peptides that target the N-terminus of FZD7 containing the KTxxxW motif region have shown the best inhibition effect, indicating that the blockade between FZD7 and DVL is a critical event to the degradation of β-catenin. The downstream β-catenin/TCF activity and expression of β-catenin target genes are decreased upon the blockade between FZD7 and DVL. This serves as a proof-of-concept that FZD7 is a potential therapeutic target in HCC. The consequence of the disruption between FZD7 and DVL upon small molecule (FJ9) treatment was also reported in lung cancer and melanoma cell lines [84]. Results showed that β-catenin/TCF activity is reduced in response to the disruption between FZD7 and DVL, leading to the suppression of tumor growth. Besides blocking the interaction between FZD7 and DVL to target cancer, Wnt signaling can also be inhibited through the blockade between Wnts and FZD receptors. Wei et al. reported that soluble FZD7 (sFZD7) interacts with Wnt3 and thereby interferes with the binding between Wnt3 and FZD receptors in HCC [85]. This results in the suppressive effect on the Wnt-induced β-catenin signaling in HCC and the downregulation of Wnt/β-catenin target genes, leading to a decrease in tumor growth. Particularly, sFZD7 could sensitize HCC cells to doxorubicin, suggesting the potential combined use of both sFZD7 and chemotherapeutic agents in HCC. Apart from targeting FZD receptors using small molecules and sFZD, the use of an anti-FZD7 antibody has also been assessed in tumorigenesis. One report has demonstrated that a monoclonal FZD7 antibody abrogated the stemness properties in FZD7-sensitive Wilm’s tumor cells [86]. Wnt/β-catenin signaling is a key pathway for maintaining cancer stemness [87]; this finding implies the use of FZD7 antibodies to target the population of tumor-initiating cells (T-ICs). Other than targeting FZD receptors, Xu et al. made use of the FZD7 promoter to target HCC through a genetic approach [88]. As FZD7 expression and FZD7 promoter activity are increased in HCC tissues, a recombinant vector with an FZD7 promoter and a Shiga-like toxin 1 (*Stx*1) gene—a toxin generated by bacteria—was constructed to evaluate the cytotoxicity of Stx1 on HCC. Data reveal that transfection of this construct induced HCC cell apoptosis while no significant effect was observed in an immortalized normal liver cell line. This piece of evidence illustrates the potential of FZD7 promoter as a gene therapy in HCC. Since FZD7 was also found to be overexpressed in dysplastic liver tissue, use of the FZD7 promoter might be beneficial in targeting early HCC development. The potential of targeting the FZD7 receptor has also been evaluated in other cancers. A study revealed that inhibitors of SIRT1/2 reduce FZD7 expression in breast cancer cell lines by decreasing β-catenin enrichment at the FZD7 promoter and that silencing FZD7 suppressed breast cancer cell migratory ability and cell growth [89]. This further suggests that the FZD7 receptor has a prominent role in cancer progression, leading to an attractive therapeutic target in cancer therapy.

Given the various degrees of similarity between the FZD receptors, it might be worth investigating the global effect of an FZD antibody against all FZD receptors. Remarkably, an anti-FZD antibody, OMP-18R5, is able to interact with 5 out of 10 FZD receptors [90]. Mechanistically, using HEK293 cells, it is postulated that OMP-18R5 blocks the interaction between Wnt3A and FZD receptors by binding to the CRD on the FZD receptors. Functionally, Wnt signaling is suppressed and this led to the decrease in tumor growth upon OMP-18R5 treatment in a mouse model. The combination treatment of OMP-18R5 and a chemotherapeutic agent showed the greatest suppressive effect on tumor growth and delayed tumor recurrence. These results ascertain the therapeutic potential of targeting FZD receptors in cancer therapy. Notably, a phase 1 trial of OMP-18R5 has been completed in patients with solid tumors (ClinicalTrials.gov identifier: NCT01345201). A phase 1b clinical trial has been completed in the study of OMP-18R5 (vantictumab) in combination with docetaxel in lung cancer patients (ClinicalTrials.gov identifier: NCT01957007). Two phase 1b trials of OMP-18R5 in combination with chemotherapeutic agents are ongoing in breast cancer and pancreatic cancer patients (ClinicalTrials.gov identifier: NCT01973309; NCT02005315). Another preclinical study revealed the adoption of OMP-54F28 (ipafricept), a truncated FZD8 receptor fused with the IgG1 Fc region, reduces Wnt signaling and tumor growth in patient-derived HCC and ovarian cancer models [91,92]. These move forward to phase 1 clinical trials. To date, a phase 1 trial of OMP-54F28 in patients with solid tumors has been completed (ClinicalTrials.gov identifier: NCT01608867) while two phase 1b trials of OMP-54F28 in combination with chemotherapeutic agents are ongoing in pancreatic cancer and ovarian cancer patients (ClinicalTrials.gov identifier: NCT02050178; NCT02092363). More relevant, a phase 1b trial of OMP-54F28 in combination with sorafenib in HCC patients has been completed recently (ClinicalTrials.gov identifier: NCT02069145). Since Wnt signaling is essential in developmental biology and adult tissue homeostasis, it is important to evaluate the adverse effects caused by the FZD-targeted therapy. Of note is that the most common related adverse events included grade 1 and 2 fatigue, vomiting, abdominal pain, constipation, diarrhea, nausea, and neutropenia for OMP-18R5, while the most common related grade 1 and 2 adverse events included dysgeusia, decreased appetite, fatigue, muscle spasms, nausea, and vomiting for patients treated with OMP-54F28 in the phase 1 clinical trials [93,94,95]. It is also worth highlighting that bone fractures and an increase in β-C-terminal telopeptide resulted in 1 out of 18 patients and 5 out of 25 patients treated with OMP-18R5 and OMP-54F28, respectively. The findings from the clinical trials suggest that targeting FZD receptors would cause the abovementioned tolerable adverse events with various degrees in bone modulation. Since there is no information about the adverse effects in liver reported in the clinical trials, this suggests that there might not be apparent adverse effects in the liver upon FZD-targeted therapy. However, liver function can further be evaluated by measuring the level of liver enzymes in patients treated with FZD-targeted therapy. Although the aforementioned clinical trials have been mostly carried out in cancer patients other than HCC, the success following completion of phase 1 trials provides invaluable information on the safety of FZD-targeted therapy in HCC patients, as well as the potential combination of FZD-targeted therapy and FDA-approved targeted therapy in HCC patients.

## 8. Future Perspectives

Wnt signaling is described as a druggable pathway in cancer. Significant efforts have been put toward inventing and evaluating drugs that target the key components along this pathway. Having said that, FZD receptors emerge as attractive targets but only a small portion of drugs have been successfully assessed in clinical trials. In other words, targeting FZD receptors as a practical solution to cancer is still underway. In view of the ongoing status of phase 1b studies of OMP-18R5 and OMP-54F28 in patients with solid tumors, the practicality of these drugs in a cancer setting awaits confirmation. While assessing their effectiveness in clinical trials, there is an urgent need to discover more potential Wnt/FZD antagonists. Koval and Katanaev reviewed existing high-throughput screening platforms to achieve a bird’s eye view of potential druggable targets along Wnt signaling [96]. It is worth noting that targeting the initiation step of Wnt signaling appears to be a better approach as the risk for adverse effects could be reduced. Conducting more high-throughput screening could profoundly shortlist the druggable targets in HCC. In particular, as FZDs belong to the GPCR family, current emerging tools for GPCR ligand screening might facilitate drug discovery in targeting the Wnt/FZD complex [97]. Apart from identifying more potential Wnt/FZD antagonists, the development of therapeutic agents is necessary. Currently, synthetic biological agents and small molecules are available to target FZD receptors. Regarding the manufacturing process, the development of small molecules appears to be a relatively cost-effective approach [98]. Still, there is scarce information on the invention of small molecules in targeting FZD receptors. Besides targeting FZD7 using FJ9, Lee et al. identified five small molecules that could specifically bind to the CRD of the FZD8 receptor; however, functional effects of these inhibitors in a cancer setting await investigation [99]. In the future, more potential Wnt/FZD antagonists shall be uncovered with the help of advanced high-throughput screening platforms and this eventually will lead to the development of biological agents or small molecules as therapeutic agents in targeting HCC.

## 9. Conclusions

Wnt/β-catenin signaling is heavily implicated in HCC progression of which FZD receptors appear to be one of the critical elements in the regulation of this pathway. The expression of multiple FZD receptors has been reported to be altered in clinical HCC tissues and deregulation of these receptors confers the aggressive phenotype of HCC, including promoting cell proliferation, metastasis, and cancer stemness. As a cell surface receptor, FZD receptors serve as attractive therapeutic targets for HCC. Small molecules and monoclonal antibodies are effective in reducing tumor growth by disrupting the interaction between FZD receptors and DVL and blocking the activation of FZD receptors in response to Wnts, respectively. Clinical trials using biological agents to target FZD receptors in cancer patient are still underway, yet the targeting of FZD receptors should show to be a potent anticancer strategy.

## Figures and Tables

**Table 1:** Summary of clinical studies of Frizzled (FZD) receptors in hepatocellular carcinoma (HCC).

Frizzled Receptors	Expression Status in HCC	Proposed/Proven Functional Significance	Reference
3/6/7	Upregulation	—Deregulation of FZD3/6/7 appeared to be a common and an early event during hepatocarcinogenesis.	[8]
1/7	Upregulation	—Expression of FZD1/7 was associated with myofibroblast activation in HCC.	[53]
2	Upregulation	—FZD2 expression was correlated with poorly differentiated HCC and late stage HCC. —HCC patients with high FZD2 expression were associated with poor recurrence-free survival. —FZD2 conferred EMT and HCC aggressiveness through activation of Wnt signaling.	[58,59]
7	Upregulation	—Deregulation of FZD7 appeared to be an early event during hepatocarcinogenesis. —Upregulation of FZD7 was associated with nuclear accumulation of wild-type β-catenin and activation of Wnt/β-catenin pathway in HCC. —Wnt3 activated Wnt/β-catenin pathway through direct binding with FZD7 in HCC.	[45,60,61]

## Abbreviations

7-TM	7-transmembrane
APC	adenomatous polyposis coli
CK1	casein kinase 1
CRD	cysteine-rich domain
EMT	epithelial-to-mesenchymal transition
FZD	Frizzled
GPCRs	G-protein coupled receptors
GSK3β GDP GTP	glycogen synthase kinase 3β Guanosine disphosphate Guanosine triphosphate
HCC	hepatocellular carcinoma
HBV	hepatitis B virus
HBx	hepatitis B X protein
HCV	hepatitis C virus
JNK	c-Jun N-terminal protein kinase
LRP 5/6	LDL receptor-related proteins 5 and 6
LEF/TCF	lymphoid enhancer factor/T cell factor
ROCK	Rho-associated kinase
T-ICs	tumor-initiating cells
SAPK	stress-activated protein kinase
sFRP	secreted frizzled-related protein
PCP	Planar cell polarity
PDZ	Psd-95/Disc large/ZO-1 homologous
